# Rudorffite Silver‐Bismuth Iodides: Emerging Eco‐Friendly Wide‐Bandgap Absorbers for Indoor Photovoltaics

**DOI:** 10.1002/smll.202510252

**Published:** 2025-11-06

**Authors:** Taeho Moon

**Affiliations:** ^1^ Department of Materials Science and Engineering Dankook University Cheonan 31116 South Korea

**Keywords:** indoor photovoltaics, rudorffite silver‐bismuth iodides, solution processing, vapor processing, wide‐bandgap absorbers

## Abstract

Indoor photovoltaics (IPVs) are poised to play a pivotal role in powering low‐consumption electronics, including wireless sensors and Internet of Things (IoT) devices, by harvesting energy from ambient light. Among emerging absorbers, silver–bismuth iodide rudorffites (Ag_x_Bi_y_I_x+3y_) have attracted increasing attention as eco‐friendly, wide‐bandgap semiconductors offering strong visible absorption, intrinsic thermal and ambient stability, and the absence of toxic Pb. Recent years are rapid progress, with indoor power conversion efficiencies reaching ≈5% under 1000 lx light‐emitting diode illumination. These advances are enabled by improved understanding of polymorphism, defect states, and charge‐carrier dynamics, coupled with innovations in film fabrication via both solution and vapor processing. Strategies such as hot‐air‐assisted crystallization, compositional tuning, and hole transport material engineering have proven particularly effective in enhancing device performance and stability. This review summarizes the crystallographic, optical, and electronic properties of Ag–Bi–I rudorffites, compares fabrication approaches, and highlights recent device demonstrations, including semi‐transparent and planar architectures. Remaining challenges—such as mitigating carrier localization, reducing deep defect densities, achieving scalable fabrication, and ensuring long‐term stability—are discussed, along with opportunities for integration into practical IPV systems. Continued research may establish rudorffites as a sustainable, commercially viable alternative to Pb‐based indoor photovoltaic technologies.

## Graphical abstract

This review highlights recent advances in rudorffite silver–bismuth iodides (Ag–Bi–I) as eco‐friendly, wide‐bandgap absorbers for indoor photovoltaics. It summarizes their crystallographic and optoelectronic properties, compares solution‐ and vapor‐processed film formation, and discusses device engineering strategies—such as hot‐air‐assisted crystallization and interface optimization—that drive efficient and stable performance in lead‐free photovoltaic devices under indoor light.

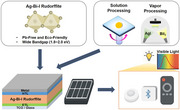

## 1 Introduction

The rapid widespread adoption of Internet of Things (IoT) devices has created a growing demand for autonomous power supply systems that can operate without wiring or external power sources. Such self‐powered systems offer advantages in ease of installation, integration, and reduced infrastructure costs. Indoor photovoltaics (IPVs) have emerged as an effective alternative, capable of providing stable power to IoT sensors and sustaining wireless communication. Compact IPV modules with areas of only a few square centimeters can supply sufficient power for many indoor devices.^[^ ^1^ ^]^

Unlike outdoor photovoltaics, IPVs utilize low‐intensity, visible‐light‐dominated illumination from artificial sources such as fluorescent lamps, light‐emitting diodes (LEDs), and halogen lights. According to the Shockley–Queisser limit, the maximum theoretical power conversion efficiency (PCE) under indoor spectra is 52–57% when the absorber bandgap is in the range of 1.8–2.0 eV. This bandgap range not only matches the emission spectrum of typical indoor light sources but also supports high open‐circuit voltages (*V*_OC_), which are crucial for powering electronic circuits or charging storage devices efficiently. Higher *V*oc reduces the number of series‐connected cells required, simplifying module design and lowering manufacturing costs. In low‐light conditions, where photocurrent is inherently reduced, minimizing recombination and leakage currents becomes a critical factor in maintaining high voltage and overall efficiency.^[^ ^2^, ^3^, ^4^ ^]^

Commercial IPVs are currently represented by amorphous silicon (bandgap ≈1.7 eV) cells from Panasonic and solid‐state dye‐sensitized cells (bandgap ≈1.74 eV) from Ambient Photonics, which are already deployed in IoT sensors, remote controls, and wearable electronics.^[^ ^5^, ^6^ ^]^ However, their *V*oc values typically remain below 1 V, limiting their energy‐harvesting potential.

Among the emerging materials for IPVs, Pb‐based halide perovskites have demonstrated exceptional efficiencies under indoor lighting.^[^ ^7^, ^8^, ^9^, ^10^, ^11^, ^12^ ^]^ Yet, the inherent toxicity of Pb presents serious environmental and regulatory challenges, especially in indoor settings. This has motivated intensive research into Pb‐free light absorbers with wide band gaps and high stability.

In this context, silver–bismuth halides with the general formula Ag_x_Bi_y_I_x+3y_ have attracted attention as promising alternatives. These materials, often referred to as rudorffite silver–bismuth iodides, feature edge‐sharing octahedral frameworks, exhibit band gaps in the range of 1.8–2.0 eV, possess high absorption coefficients, and maintain excellent structural stability under ambient conditions. Early demonstrations confirmed their photovoltaic activity, although efficiencies under standard illumination were initially low. More recently, advances in compositional tuning (e.g., Ag:Bi ratio optimization) and processing innovations have enabled indoor device PCEs of up to 5% under 1000 lx illumination, with *V*_OC_ reaching 0.8 V or higher under 1 sun—representing a meaningful milestone for Pb‐free IPVs.^[^ ^13^ ^]^

This review examines the current state of rudorffite Ag–Bi–I compounds as wide‐bandgap absorbers for IPVs. We survey their crystallographic structures and fundamental optoelectronic properties, which underpin their functionality. Two major fabrication pathways—solution and vapor processing—are compared, emphasizing their influence on film morphology and device performance. We then highlight recent demonstrations of indoor devices, focusing on performance and long‐term operational stability. Finally, we provide an outlook on the challenges and opportunities for integrating silver–bismuth iodide rudorffites into practical, environmentally friendly IPVs.

## 2 Structural and Optoelectronic Properties

### 2.1 Crystal Structure and Polymorphism

Ag–Bi–I rudorffites form a family of compounds exhibiting structural polymorphism, but they share a common feature: they are double halide salts consisting of Ag⁺, Bi^3^⁺, and I^−^ in fixed ratios. A prototypical member of this family is AgBiI_4_, which crystallizes in structures related to rudorffite phases. These structures are characterized by closely packed iodide layers and ordered occupation of octahedral sites by Ag⁺ and Bi^3^⁺ cations, forming edge‐sharing AgI_6_ and BiI_6_ octahedra. This structural motif stands in contrast to the 3D corner‐sharing PbI_6_ framework found in lead halide perovskites.

**Figure 1:**
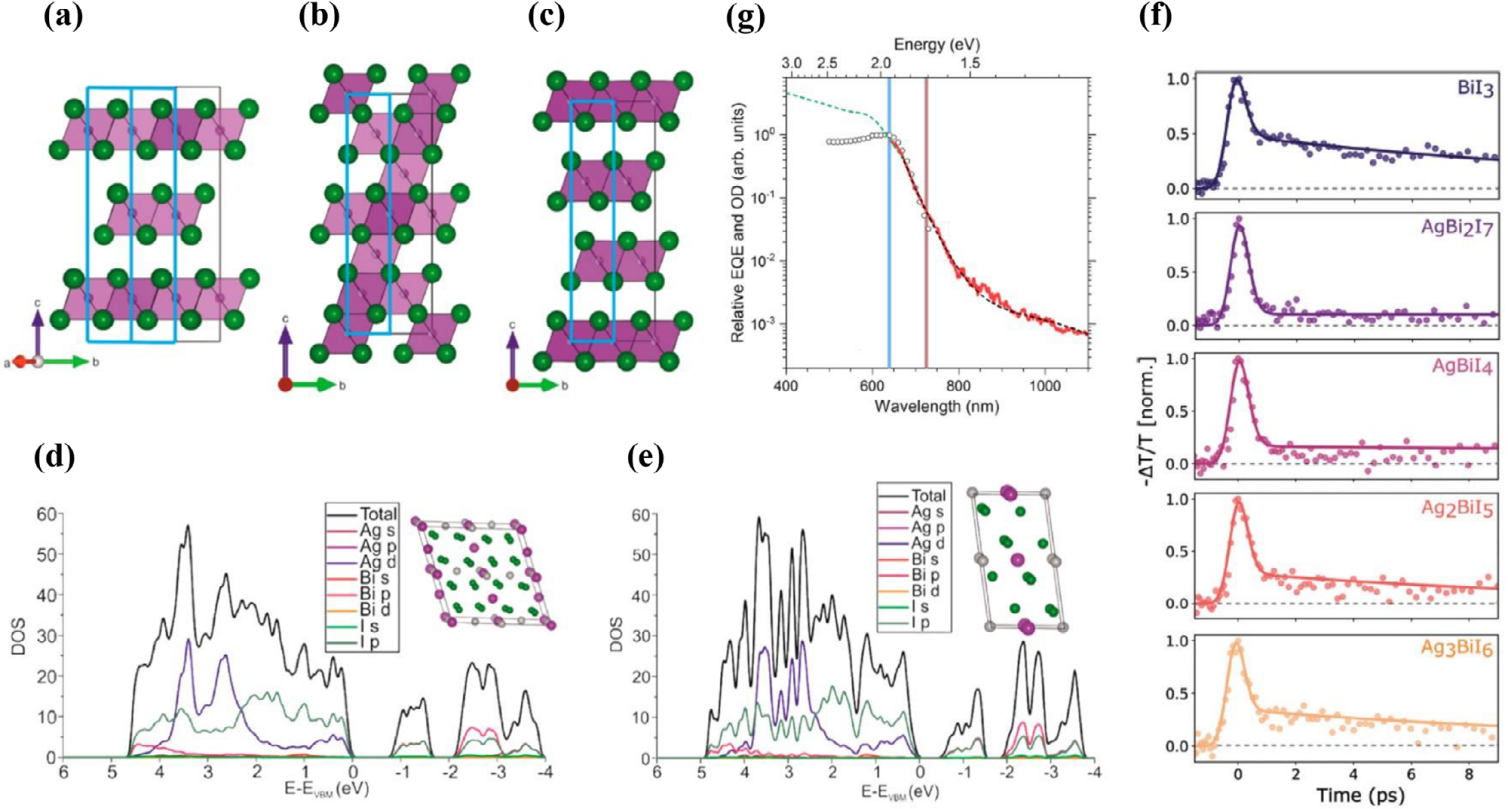
Structural and Optoelectronic Properties. a–c) Crystal structures of BiI_3_ and two polymorphs of AgBiI_4_. The green spheres represent iodide (I) atoms located at the octahedral vertices. In (a), Bi atoms occupy the centers of the BiI_6_ octahedra, whereas in (b,c), the split circles denote mixed Ag/Bi occupancy at the cation sites. a) Layered BiI_3_ with hexagonal close‐packed iodide layers and partially occupied Bi^3^⁺ sites. b) Defect‐spinel‐type–type AgBiI_4_ with alternating Ag⁺/Bi^3^⁺ occupancy in a cubic close‐packed lattice. c) CdCl_2_‐type AgBiI_4_ showing layered cation ordering. Blue boxes represent the CdCl_2_‐type reference unit cell for comparison. d,e) DOS for the (d) defect‐spinel and (e) CdCl_2_‐type structures of AgBiI_4_, corresponding to their lowest‐energy polymorphs. (a–e) Reproduced with permission.^[^ ^14^ ^]^ Copyright 2017, ACS Publications. f) Normalized photoinduced THz conductivity transients (−ΔT/T) of (AgI)_x_(BiI_3_)_y_ thin films measured by optical‐pump THz‐probe spectroscopy. Reproduced with permission.^[^ ^17^ ^]^ Copyright 2024, Wiley. g) Fourier‐transform photocurrent spectroscopy (FTPS) spectrum (solid red line) of an AgBi_2_I_7_ thin film on an interdigitated ITO substrate, compared with steady‐state absorption (dashed green line) and monochromator‐based photocurrent data (open circles). Reproduced with permission.^[^ ^16^ ^]^ Copyright 2023, ACS Publications.

**Figure** **1** summarizes the representative crystal structures of BiI_3_ and the two known polymorphs of AgBiI_4_, emphasizing the relationship between iodide sublattice packing and cation ordering.^[^ ^14^ ^]^ BiI_3_ crystallizes in a layered structure based on hexagonal close‐packed (HCP, ABA) iodide layers, with edge‐sharing BiI_6_ octahedra partially occupied by Bi^3^⁺ (2/3 occupancy) and 1/3 vacant, alternating with fully empty interlayers (Figure 1a). In contrast, AgBiI_4_ adopts a cubic close‐packed (CCP, ABC) iodide sublattice and can form two distinct polymorphs:

1. A defect‐spinel–type cubic structure (Figure 1b), where Ag⁺ and Bi^3^⁺ cations occupy interstitial octahedral sites in alternating layers (3/4 and 1/4 occupancy, respectively), forming a 3D network of edge‐sharing octahedra.
2. A CdCl_2_‐type rhombohedral layered structure (Figure 1c), in which cations fully occupy every other plane, separated by completely vacant cation layers.

Density functional theory (DFT) calculations suggest that these two polymorphs are nearly degenerate in energy, differing by only ≈18 meV per formula unit. This small energy difference implies that both phases are accessible and that their formation may depend sensitively on processing conditions.

Beyond AgBiI_4_, several Ag–Bi–I compounds have been explored, exhibiting distinct structural features depending on their Ag:Bi ratios.^[^ ^15^ ^]^ Ag‐rich phases such as Ag_2_BiI_5_ and Ag_3_BiI_6_ tend to adopt the rhombohedral structure, while Bi‐rich compositions like AgBi_2_I_7_ more commonly exhibit the defect spinel‐type cubic structure. As the Ag content increases, a systematic expansion of the unit cell is observed, attributed to the larger ionic radius of Ag⁺ relative to Bi^3^⁺.

### 2.2 Optical Bandgap and Absorption Properties

The photovoltaic performance of a material is fundamentally governed by its optical bandgap and absorption characteristics. A representative optical study on Ag–Bi–I compounds—including Ag_3_BiI_6_, Ag_2_BiI_5_, AgBiI_4_, and AgBi_2_I_7_—reported indirect band gaps in the range of 1.66–1.80 eV and direct transition onsets ≈1.93–2.00 eV.^[^ ^16^ ^]^ While both transitions are optically allowed, the absorption near the indirect transition is relatively weak, rendering these materials effectively wide‐bandgap semiconductors, with significant absorption occurring above ≈1.9 eV. These values align well with the indoor lighting spectrum.

Despite compositional variation, no clear trend in bandgap was observed with respect to the AgI:BiI_3_ ratio. This has been rationalized by density‐of‐states (DOS) calculations, which reveal that the band‐edge states are primarily composed of Bi 6p and I 5p orbitals—similar to BiI_3_—with only minor contribution from Ag. DFT calculations for AgBiI_4_ confirm this picture: in both the defect‐spinel and CdCl_2_‐type polymorphs, the valence band maximum (VBM) originates mainly from I 5p states, while the conduction band minimum (CBM) arises from hybridized Bi 6p and I 5p orbitals (Figure 1d,e).^[^ ^14^ ^]^ The negligible involvement of Ag in the band‐edge states suggests that variations in Ag content have a limited influence on optical transition energies.

Furthermore, the close similarity in DOS profiles between the two structural variants of AgBiI_4_ implies that its optoelectronic properties are robust against structural polymorphism. In addition to having a favorable bandgap, Ag–Bi–I thin films exhibit high optical absorption coefficients—on the order of 10⁵ to 10⁶ cm^−1^ in the visible range—comparable to those of Pb‐based perovskites, highlighting their promise as PV‐free IPV absorbers.^[^ ^14^ ^]^

### 2.3 Carrier Localization and Defect States

A defining photophysical feature of Ag–Bi–I rudorffites is their strong carrier localization behavior, driven by structural and energetic disorder. Time‐resolved spectroscopy studies—including terahertz conductivity and transient absorption—reveal that photogenerated carriers undergo ultrafast self‐localization on sub‐picosecond timescales. This phenomenon is attributed to strong electron–phonon coupling and local lattice disorder, which promote small‐polaron formation and reduce carrier mobility.

To probe the influence of chemical composition, optical‐pump THz‐probe spectroscopy was performed on (AgI)_x_(BiI_3_)_γ_ thin films (Figure 1f).^[^ ^17^ ^]^ All compositions exhibited a sharp drop in photoconductivity within ≈1 ps after excitation, reflecting a rapid transition from delocalized to localized transport. Notably, this mobility loss dominates the decay dynamics, as transient absorption reveals a persistent carrier population following the conductivity drop. The degree of localization is composition‐dependent: BiI_3_ shows a gradual decay, whereas Ag‐deficient phases such as AgBi_2_I_7_ exhibit a more abrupt mobility loss.

Further insights were obtained via Fourier‐transform photocurrent spectroscopy (FTPS) on AgBi_2_I_7_ (Figure 1g).^[^ ^16^ ^]^ The FTPS spectrum displays a broad sub‐bandgap photocurrent response extending to ≈1100 nm, indicating a high density of trap states. Modeling with an Urbach tail and Gaussian defect distribution yields an Urbach energy of 70 meV and a deep‐level defect ≈0.6 eV below the band edge. These features confirm that non‐radiative recombination through deep traps and energetic disorder is intrinsic to the material. Therefore, overcoming these limitations will likely require compositional engineering strategies to suppress polaron formation and reduce trap‐state densities.

Complementary structural and spectroscopic studies have provided additional insight into how compositional tuning can alleviate such defect‐induced localization. Chang et al. synthesized a series of Ag–Bi–I rudorffite compounds via a gas–solid phase reaction and systematically investigated their local electronic structures using X‐ray photoelectron spectroscopy (XPS), X‐ray absorption near‐edge structure (XANES), and extended X‐ray absorption fine structure (EXAFS) analyses.^[^ ^18^ ^]^ As the Ag/Bi ratio increased, partial reduction and delocalization of Ag⁺ ions occurred, accompanied by the occupation of vacant tetrahedral sites and local rearrangement of Ag–I coordination. This process led to electron‐density redistribution and suggested the formation of more continuous out‐of‐plane (c‐axis) transport pathways. The authors interpreted these changes as a structural–electronic mechanism by which delocalized Ag could fill vacancy defects and thereby mitigate trap‐mediated recombination.

### 2.4 Thermal, Light, and Ambient Stability

In addition to optoelectronic performance, environmental and operational stability are critical for photovoltaic applications. Ag–Bi–I rudorffites such as AgBiI_4_ demonstrate superior thermal stability compared to many hybrid perovskites, remaining structurally intact up to ≈90 °C in the dark—well above typical indoor operating temperatures.^[^ ^14^ ^]^

However, their photostability exhibits a more complicated behavior. Like other halide materials, AgBiI_4_ is susceptible to light‐induced degradation, particularly under short‐wavelength irradiation (<600 nm) and in the presence of ambient air. A representative study reported partial photodecomposition into crystalline AgI after exposure to the full solar spectrum (100 mW cm^−2^), while long‐wavelength light (600–761 nm) caused no significant structural change. This suggests that AgBiI_4_ is significantly more stable under indoor illumination due to the markedly reduced photon flux across the high‐energy spectral range, including UV and parts of the blue region. Encapsulation further enhances photostability, with protected samples showing marked resistance to degradation even under prolonged full‐spectrum exposure. These observations suggest that the degradation of AgBiI_4_ arises from a combined influence of light and ambient factors such as oxygen and moisture, rather than from photon exposure alone. The process likely involves photoexcited charge carriers that facilitate redox interactions between iodide and metal species, leading to partial AgI formation under high‐energy illumination. While the detailed mechanism remains to be clarified, further in situ spectroscopic or theoretical studies would be valuable for elucidating the photo‐redox pathways involved.

Moreover, AgBiI_4_ exhibits excellent ambient stability in the absence of light. Samples stored under ambient conditions in the dark retain their structural integrity for at least six weeks, indicating that these materials are inherently resistant to moisture and oxygen‐induced degradation—a major advantage over many hybrid perovskites. These findings collectively indicate that Ag–Bi–I rudorffites are both thermally and environmentally stable, especially under the low‐intensity light conditions found in indoor environments.

### 2.5 Structural Connectivity and Defect Tolerance

Lead‐free halide absorbers exhibit diverse crystal architectures that can be arranged along a structural‐dimensionality continuum, from fully connected 3D perovskites to isolated 0D clusters. At one extreme, 3D perovskites feature corner‐sharing octahedra that enable isotropic charge transport but suffer from chemical instability. Double perovskites (e.g., Cs_2_AgBiBr_6_) retain this framework with ordered B/B′ cation alternation, improving lattice stability at the expense of wider and often indirect band gaps. Rudorffite‐type compounds, such as Ag–Bi–I and Ag–Sb–I families, occupy an intermediate structural regime between 3D perovskites and 0D Bi halides. Their edge‐sharing octahedral networks maintain partial connectivity but also exhibit cation–vacancy ordering and local distortions that limit long‐range electronic coupling. Beyond this regime, 1D chain‐type halides (e.g., CuBiI_4_) and 0D layered or molecular Bi halides (e.g., A_3_Bi_2_I_9_) exhibit progressively weaker connectivity and stronger carrier confinement.

The degree of octahedral connectivity and metal–halide bonding character strongly influences defect tolerance. Bi‐based halides typically show pronounced carrier localization because limited orbital overlap and strong phonon coupling confine wavefunctions within individual octahedra, leading to deeper trap states and shorter diffusion lengths.^[^ ^19^ ^]^ As structural dimensionality decreases from 3D to lower‐dimensional frameworks, the band‐edge dispersion becomes flatter with larger carrier masses and deeper defect levels, whereas strongly corner‐sharing lattices with pronounced antibonding s–p coupling (e.g., Pb–I) exhibit more dispersive bands, delocalized carriers, and shallow defect states.^[^ ^20^ ^]^

Collectively, these experimental and theoretical insights highlight that both structural connectivity and orbital coupling govern the spatial localization of defect states and thus the intrinsic defect tolerance of halide absorbers. Within this framework, the intermediate connectivity and partial vacancy ordering of Ag–Bi–I rudorffites likely result in a defect tolerance lower than that of 3D perovskites but higher than that of 0D Bi halides, consistent with their structurally intermediate nature.

Overall, Ag–Bi–I rudorffites can be regarded as wide‐bandgap semiconductors that combine strong visible absorption and robust environmental stability with eco‐friendly, lead‐free composition, rendering them promising candidates for IPV applications. Nevertheless, their photovoltaic performance remains limited by rapid carrier localization and high trap densities arising from intrinsic lattice disorder and restricted orbital coupling. These inherent constraints motivate recent efforts to improve film quality and reduce defect densities through controlled processing and compositional tuning, as discussed in the following section.

## 3 Solution‐Processed Rudorffite Absorbers

### 3.1 Solvent‐Controlled Crystallization

Building on the stability and electronic characteristics discussed above, recent studies have focused on improving film quality and reducing defect density through solution‐processing strategies. Such approaches aim to mitigate carrier trapping and localization—identified as intrinsic limitations of Ag–Bi–I rudorffites—by optimizing crystallization pathways and morphology control.

Solvent engineering plays a crucial role in directing the nucleation behavior, crystallization pathway, and resulting morphology of Ag–Bi–I rudorffite thin films. By controlling solvent type, anti‐solvent treatment, and intermediate adduct formation, researchers have achieved improved film coverage, reduced defect density, and enhanced device performance.

**Figure 2:**
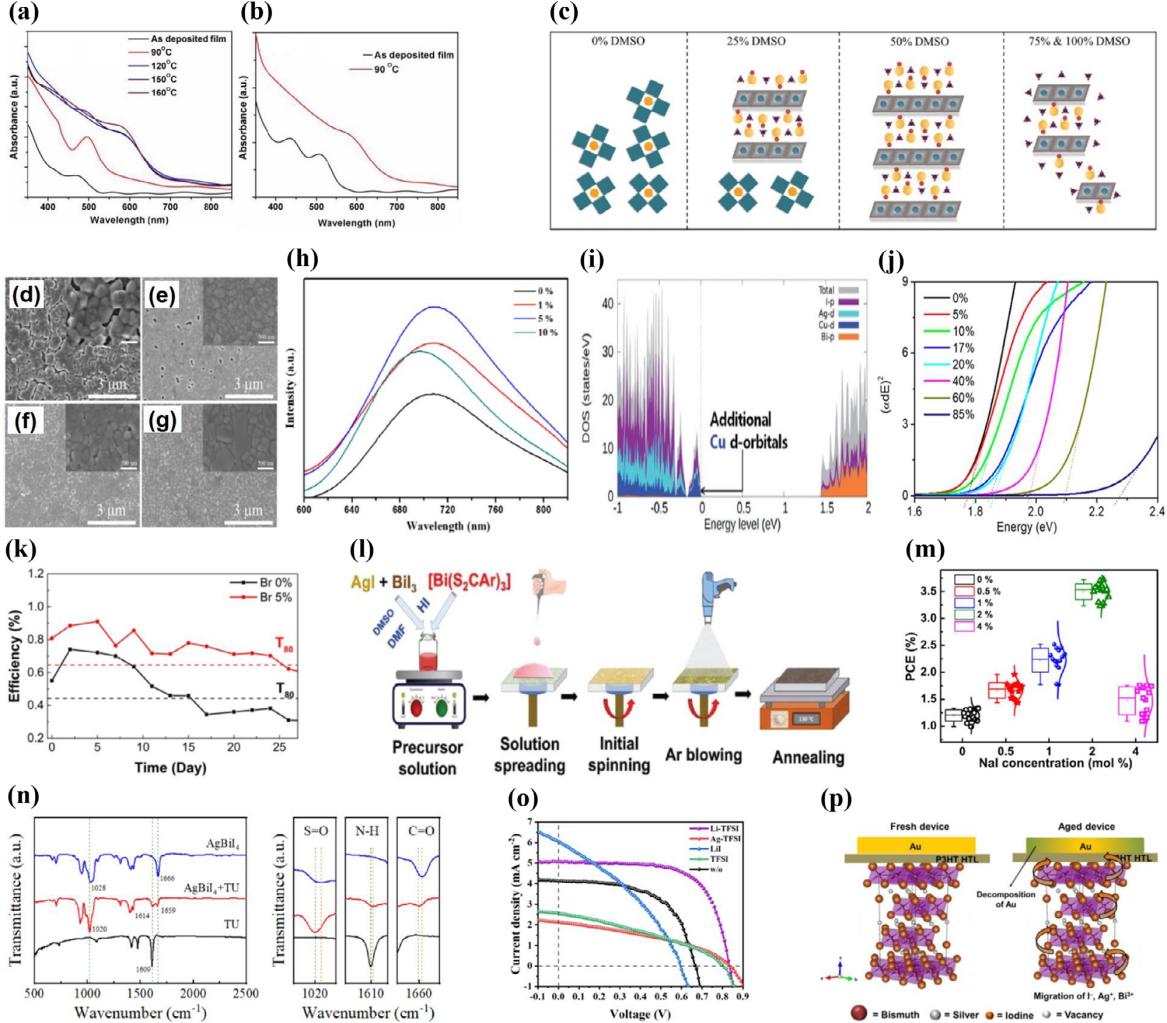
Solution‐Processed Rudorffite Absorbers. a,b) UV–vis absorption spectra of AgBi_2_I_7_ thin films obtained at different temperatures using (a) n‐butylamine and (b) DMSO as solvents. a,b) Reproduced with permission.^[^ ^19^ ^]^ Copyright 2019, Royal Society of Chemistry. c) Schematic illustration of the effect of DMSO volume ratio in DMF/DMSO precursor solutions on the formation of intermediate phases during film crystallization. Reproduced with permission.^[^ ^22^ ^]^ Copyright 2021, ACS Publications. d–g) SEM images of the AgBiI_4_ films fabricated (d) without the antisolvent treatment and (e–g) with the antisolvent treatments of (e) toluene, (f) chlorobenzene, and (g) isopropanol, respectively. d–g) Reproduced with permission.^[^ ^23^ ^]^ Copyright 2022, Royal Society of Chemistry. h) PL spectra of AgBiI_4_ thin films with varying Cs incorporation levels (0, 1, 5, and 10%). Reproduced with permission.^[^ ^24^ ^]^ Copyright 2021, ACS Publications. i) Orbital‐decomposed partial density of states in Cu‐doped Ag_2_BiI_5_. Reproduced with permission.^[^ ^25^ ^]^ Copyright 2021, Royal Society of Chemistry. j) Tauc plots for AgBi_2_(I_1‐x_Br_x_)_7_ thin films with varying Br contents. Reproduced with permission.^[^ ^26^ ^]^ Copyright 2019, ACS Publications. k) Stability‐test results of AgBi(I,Br)_4_ solar cells with 0% and 5% Br, measured under ambient conditions without encapsulation. Reproduced with permission.^[^ ^27^ ^]^ Copyright 2024, Wiley. l) Schematic illustration of the fabrication procedure for depositing Ag_a_Bi_b_I_a+3b−2x_S_x_ films. Reproduced with permission.^[^ ^28^ ^]^ Copyright 2019, Wiley. m) Dependence of the PCE of AgBiI_4_ devices on NaI concentration. Reproduced with permission.^[^ ^29^ ^]^ Copyright 2025, ACS Publications. n) FTIR spectra of TU, AgBiI_4_, and AgBiI_4_–TU films, with an expanded fingerprint region highlighting the S═O, C═O, and N─H stretching vibrations. Reproduced with permission.^[^ ^30^ ^]^ Copyright 2025, ACS Publications. o) *J–V* curves of AgBiI_4_ devices with different additives. Reproduced with permission.^[^ ^31^ ^]^ Copyright 2019, ACS Publications. p) Schematic illustration of the degradation mechanism of an Ag_3_BiI_6_ device under ambient atmosphere. Reproduced with permission.^[^ ^32^ ^]^ Copyright 2022, Wiley.

Solvent coordination strength strongly influences crystallization dynamics. For example, dimethyl sulfoxide (DMSO), with relatively weak coordination ability, enables low‐temperature crystallization of AgBi_2_I_7_ at 90 °C, whereas strongly coordinating n‐butylamine requires annealing above 150 °C for complete phase conversion.^[^ ^21^ ^]^ As shown in **Figure** **2**a,b, DMSO‐based films form with minimal intermediate adducts, yielding smooth morphology, higher PCE, and improved environmental stability.

Mixed‐solvent ratios also affect intermediate phase formation and growth kinetics. As illustrated in Figure 2c, adding 25–50% DMSO to N,N‐dimethylformamide (DMF) promotes the formation of a BiI_3_–DMSO–AgI intermediate complex, which suppresses premature nucleation and enables larger grains with better coverage.^[^ ^22^ ^]^ Optimal morphology is achieved at 50% DMSO, producing dense, pinhole‐free films, whereas ≥75% DMSO causes dewetting and poor adhesion due to its hydrophobicity.

Anti‐solvent treatment provides another handle for morphology control. As shown in Figure 2d–g, isopropanol (IPA) produces smoother films with enlarged grains and fewer pinholes than toluene or chlorobenzene, owing to its higher miscibility with DMF/DMSO and moderate boiling point.^[^ ^23^ ^]^ These results highlight that solvent–anti‐solvent selection, guided by polarity, boiling point, and compatibility, is critical for achieving high‐quality Ag–Bi–I films.

### 3.2 Cation Doping Strategies

Cation doping is an effective route to enhance the optoelectronic properties and stability of Ag–Bi–I absorbers. In particular, Cs incorporation has a pronounced influence on the crystallization and film formation of AgBiI_4_, producing compact, pinhole‐free films with smoother surfaces and improved crystallinity. Moderate Cs addition (5 mol%) promotes more uniform grain growth and suppresses defect formation during solution processing, leading to enhanced photoluminescence intensity, reduced trap‐state density, and higher carrier mobility, as shown in Figure 2h.^[^ ^24^ ^]^ However, excessive Cs incorporation (10 mol%) leads to the formation of secondary phases such as Cs_3_Bi_2_I_9_, which deteriorate the film quality and device performance. Consequently, the power conversion efficiency increases from 0.95 to 1.33% for mesoporous and from 0.98 to 1.38% for planar architectures, corresponding to an improvement of ≈40% compared with undoped films.

Similarly, partial substitution of Ag⁺ with Cu⁺ in Ag_2_BiI_5_ preserves the crystal structure and bandgap while enhancing light absorption and carrier injection efficiency.^[^ ^25^ ^]^ DFT calculations (Figure 2i) show that Cu introduces additional valence‐band states through hybridization with I 5p orbitals, increasing valence–conduction band overlap. An optimal 2.5 mol% Cu content improved short‐circuit current density (*J*_SC_) and raised PCE by ≈25% relative to the pristine counterpart.

### 3.3 Anion Substitution (Halide and Chalcogenide)

Partial substitution of I^−^ with other anions such as Br^−^ or S^2−^ can effectively tune the band structure, morphology, and stability of Ag–Bi–I‐based absorbers. Br incorporation into AgBi_2_I_7_ via solution processing induces a systematic blue shift in the absorption onset, widening the direct bandgap from ≈1.78 eV (0% Br) to ≈2.28 eV (85% Br) (Figure 2j).^[^ ^26^ ^]^ DFT attributes this shift to Br 4p orbitals contributing more strongly to the valence band maximum than I 5p.

In AgBiI_4_, ≈5% Br enlarges grain size and forms dense, pinhole‐free films with reduced trap density, evidenced by stronger PL and longer carrier lifetimes.^[^ ^27^ ^]^ These improvements extend device stability, with unencapsulated 5% Br devices retaining 80% of initial efficiency for ≈26 days, compared with ≈15 days for Br‐free cells (Figure 2k). However, >10% Br promotes BiBr_3_ secondary phases and pinholes, degrading performance.

Sulfide incorporation offers an alternative anion substitution route.^[^ ^28^ ^]^ Replacing I^−^ with S^2−^ narrows the bandgap by ≈0.1 eV and upshifts the valence band edge by up to 0.3 eV, improving energy‐level alignment with HTMs. As shown in Figure 2l, Ag_3_BiI_5.92_S_0.04_ films, fabricated via gas‐assisted spin coating of AgI, BiI_3_, and Bi(S_2_CAr)_3_ precursors, achieved a PCE of 5.56%—27% higher than the sulfide‐free control—while retaining >90% efficiency after 45 days.

### 3.4 Additive Engineering Approaches

Additive incorporation into Ag–Bi–I precursor solutions is a versatile strategy to improve film formation, suppress defects, and enhance device efficiency. Additives such as alkali salts, Lewis bases, and ionic species influence crystallization dynamics, carrier transport, and interfacial quality.

In one study, 2 mol% NaI was introduced into AgBiI_4_ precursor solutions to promote grain growth and reduce surface defects without altering the crystal structure.^[^ ^29^ ^]^ Photoluminescence measurements showed higher emission intensity and longer carrier lifetime, indicating suppressed non‐radiative recombination. As shown in Figure 2m, the PCE increased from ≈1.3% (0%) to ≈3.7% at 2 mol% NaI before declining at higher concentrations due to defect formation.

Thiourea (TU), a Lewis base additive, coordinates with metal halides to retard crystallization, yielding denser films with improved electronic quality.^[^ ^30^ ^]^ FTIR spectra (Figure 2n) revealed shifts in S═O (≈1020 cm^−1^), N─H (≈1610 cm^−1^), and C═O (≈1660 cm^−1^) vibrations, confirming coordination bonding. TU incorporation enhanced light absorption, reduced charge recombination, and improved carrier extraction, increasing PCE by ≈70% compared with the control.

Lithium bis(trifluoromethanesulfonyl)imide (Li‐TFSI) was also reported to promote uniform crystal growth through the strongly coordinating TFSI^−^ anion, producing pinhole‐free films without altering the crystal structure.^[^ ^31^ ^]^ As shown in Figure 2o, Li‐TFSI delivered higher *V*_OC_ and FF than other tested additives (Ag‐TFSI, LiI, TFSI), achieving a PCE of 2.80%. However, excessive loading reduced uniformity and performance.

Collectively, these results demonstrate that the choice and concentration of additives critically affect phase purity, morphology, carrier dynamics, and ultimately the photovoltaic performance of Ag–Bi–I‐based devices.

### 3.5 Ion Migration and Degradation Mechanisms

Recent studies have revealed that Ag_3_BiI_6_ exhibits a unique triple‐ion migration involving Ag⁺, Bi^3^⁺, and I^−^, which critically impacts device stability under ambient conditions.^[^ ^32^ ^]^ This phenomenon, absent in AgBi_2_I_7_ and Ag_2_BiI_5_, is strongly influenced by thin‐film processing. Antisolvent‐processed Ag_3_BiI_6_ films contain substantial residual AgI and interfacial voids, accelerating ion migration through thin, dopant‐free HTMs and causing Au electrode corrosion with rapid performance loss. X‐ray photoelectron spectroscopy confirms the migration of all three ionic species to the electrode interface, accompanied by Au oxidation and reduction of Ag⁺ and Bi^3^⁺ to their metallic states. Density functional theory calculations indicate that Ag_3_BiI_6_ decomposition into AgI and BiI_3_ is thermodynamically favorable (ΔH = –0.053 eV), facilitating ion release. As illustrated in Figure 2p, this process involves the concerted migration of I^−^, Ag⁺, and Bi^3^⁺ toward the Au electrode, leading to electrode degradation. Mitigation strategies include minimizing residual AgI, optimizing HTL thickness, and adopting more inert electrodes.

Overall, solution processing offers a versatile route for fabricating Ag–Bi–I rudorffite absorbers, enabling substantial improvements in film quality, efficiency, and operational stability. Compared with vapor‐processed films, solution‐processed absorbers generally exhibit higher crystallinity and larger grain sizes owing to solvent‐mediated recrystallization, yet their large‐area uniformity and scalability remain limited. Wet processing can also introduce residual organic species, which may influence film composition and long‐term stability. Nevertheless, the ability to tune stoichiometry and microstructure through solvent, additive, and precursor chemistry makes the solution route highly effective for compositional exploration and defect passivation, while motivating the continued development of vapor‐phase techniques that provide intrinsic advantages in film uniformity and scalability.

## 4 Vapor‐Processed Rudorffite Absorbers

### 4.1 Overview

Building on the progress and remaining limitations of solution processing discussed above, vacuum‐based approaches offer a promising alternative for achieving large‐area film uniformity and scalability. Vacuum deposition techniques enable the fabrication of dense layers with precisely controlled thickness and are well‐suited for large‐area, multilayer device architectures.^[^ ^33^, ^34^, ^35^ ^]^ Although vapor‐processed thin films often exhibit slightly lower crystallinity than their solution‐processed counterparts, they provide clear advantages such as solvent‐free processing and excellent compatibility with scalable manufacturing. Three primary approaches have been explored: dual‐source evaporation, sequential evaporation, and single‐source ablation. While the first two rely on the thermal evaporability of metal halides such as AgI and BiI_3_, the latter involves the direct ablation of pre‐synthesized Ag–Bi–I compounds, offering a simplified single‐source route.

### 4.2 Dual‐Source Thermal Evaporation

In dual‐source evaporation, separate crucibles loaded with AgI and BiI_3_ are co‐evaporated in a vacuum chamber to deposit Ag–Bi–I thin films.^[^ ^36^ ^]^ The stoichiometry of the resulting films is controlled by adjusting the relative deposition rates of the two sources, enabling the formation of compositions such as AgBi_2_I_7_, AgBiI_4_, and Ag_2_BiI_5_. Post‐deposition annealing enhances crystallinity, drives phase formation, and removes residual secondary phases present in the as‐deposited films.

Notably, the annealing conditions—particularly the atmosphere and cooling rate—strongly influence the final crystal structure and microstructure. During annealing, partial evaporation of volatile BiI_3_ shifts the film composition toward Ag‐rich stoichiometries, which has been suggested to involve vacancy‐assisted Ag migration and lattice rearrangement. In contrast, annealing under a BiI_3_‐rich atmosphere compensates for Bi loss and stabilizes the rhombohedral phase. The cooling rate was further examined for films deposited with an initial AgI:BiI_3_ ratio of approximately 0.6, corresponding to a near‐stoichiometric AgBiI_4_ composition. Rapid quenching after annealing at 180 °C under N_2_ preserves the metastable cubic phase by freezing this configuration, whereas slow cooling (≈4 °C min^−1^) allows additional BiI_3_ volatilization and atomic rearrangement, leading to reversion to the thermodynamically favored rhombohedral structure. These observations indicate that the interplay between deposition ratio, annealing atmosphere, and cooling kinetics governs the compositional evolution and phase stability of Ag–Bi–I thin films.

**Figure 3:**
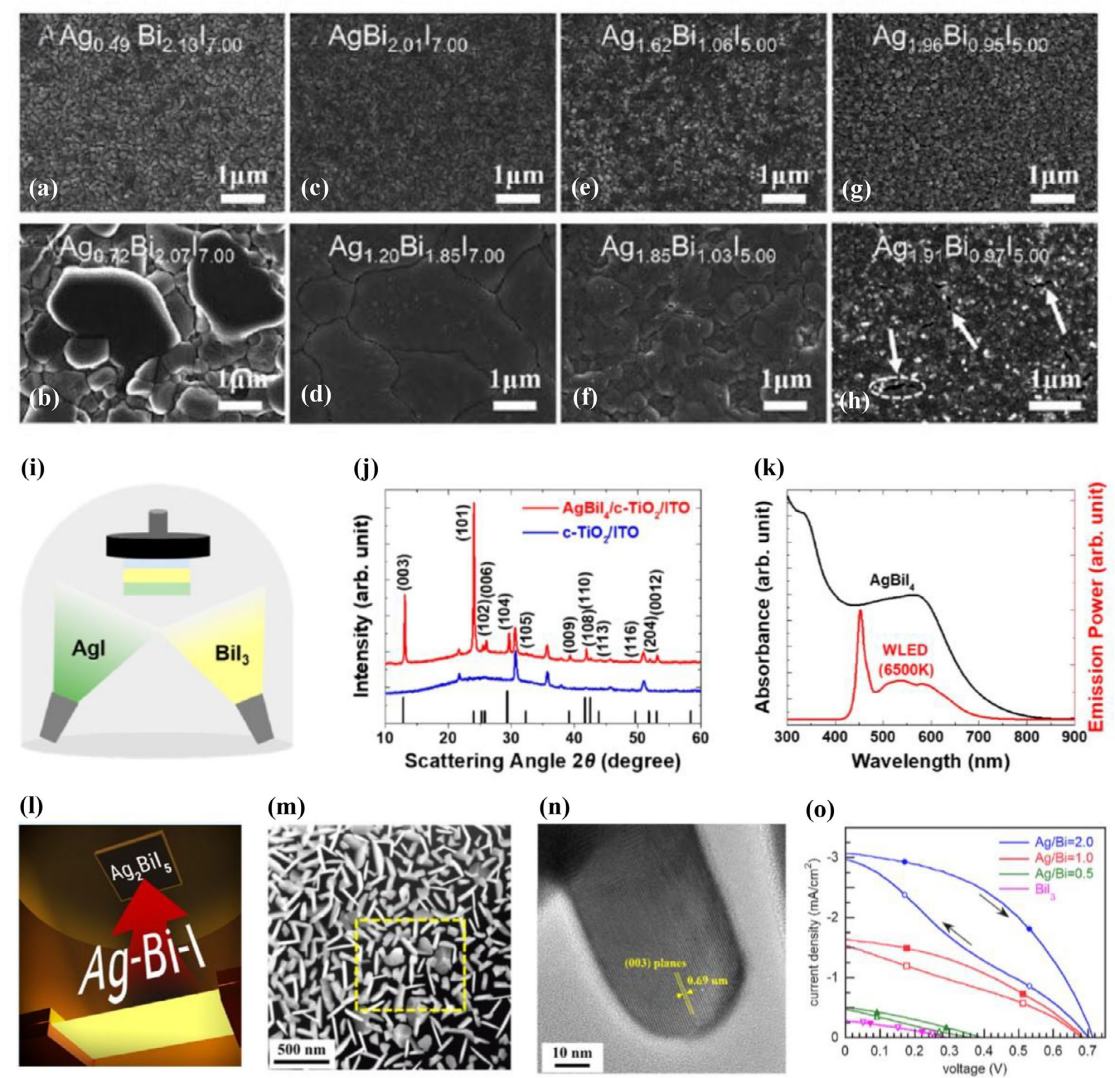
Vapor‐Processed Rudorffite Absorbers. a–h) SEM images of Ag–Bi–I thin films deposited by dual‐source evaporation with varying AgI:BiI_3_ ratios: a,b) 0.2, c,d) 0.6, e,f) 0.8, and g,h) 1.2. Top: as‐deposited; bottom: after annealing at 180 °C for 15 min. a–h) Reproduced with permission.^[^ ^36^ ^]^ Copyright 2019, Royal Society of Chemistry. i–k) i) Schematic illustration of the sequential thermal evaporation process; j) XRD pattern of a AgBiI_4_ film on c‐TiO_2_/ITO, with reference peaks for Ag_2_BiI_5_; k) Absorbance of AgBiI_4_ and WLED emission spectrum. i–k) Reproduced with permission.^[^ ^37^ ^]^ Copyright 2024, ACS Publications. l–o) l) Illustration of single‐source thermal ablation; m) Scanning transmission electron microscopy with high‐angle annular dark‐field (STEM‐HAADF) image of a film from an Ag/Bi = 1.0 precursor; n) HRTEM image of a BiI_3_ platelet showing (003) planes; o) *J*–*V* curves of solar cells from various Ag/Bi ratios under 1 sun. l–o) Reproduced with permission.^[^ ^38^ ^]^ Copyright 2025, ACS Publications.

Bi‐rich films (e.g., AgBi_2_I_7_) tend to transform into cubic phases upon annealing, whereas Ag‐rich compositions (e.g., Ag_2_BiI_5_) typically retain a rhombohedral structure. AgBiI_4_, which lies between these extremes, can crystallize into either phase depending on subtle variations in composition and thermal history. Grain size and film morphology also vary significantly with composition (**Figure** **3**a–h). Ag‐rich films exhibit large grains (>200 nm, reaching up to 3 µm) with smooth, pinhole‐free surfaces (Figure 3b,d), while AgBiI_4_ films show uniform morphology with average grain sizes ≈300 nm (Figure 3f). In contrast, Bi‐rich films display smaller grains (<100 nm) and frequent pinholes, resulting in less compact coverage (Figure 3h).

This study presents the first demonstration of solar cells employing vacuum‐deposited Ag–Bi–I absorbers in a planar architecture. Despite modest PCEs (<1%) and limited *J*_SC_ (≈2.4 mA cm^−2^) under 1 sun, the devices achieved *V*_OC_ values up to 0.84 V—among the highest reported for Ag–Bi–I systems. The observed *J–V* hysteresis suggests the presence of interfacial barriers or charge‐trapping effects that require further optimization. Nevertheless, this proof‐of‐concept highlights the viability of evaporated Ag–Bi–I materials for future integration into tandem or advanced thin‐film photovoltaic configurations.

### 4.3 Sequential Evaporation

Compared to co‐evaporation, sequential evaporation offers advantages in achieving targeted stoichiometry, as the overall composition can be precisely controlled by adjusting the individual layer thicknesses. This approach also helps suppress compositional fluctuations that may arise from differences in the vapor pressures or sticking coefficients of AgI and BiI_3_ during co‐deposition. In sequential evaporation, BiI_3_ and AgI are deposited in a defined layered sequence, followed by thermal annealing to induce interdiffusion and phase formation (Figure 3i).^[^ ^37^ ^]^

X‐ray diffraction (XRD) confirms the successful formation of crystalline AgBiI_4_ with a rhombohedral rudorffite structure, while energy‐dispersive X‐ray spectroscopy (EDS) reveals near‐stoichiometric Ag:Bi:I ratios and minimal secondary phases (Figure 3j). UV–vis absorption spectra indicate a bandgap of ≈1.89 eV, consistent with the expected range for wide‐bandgap Ag–Bi–I materials (Figure 3k). The potential application of such vapor‐processed AgBiI_4_ films in semitransparent devices for indoor photovoltaics will be discussed in the following section.

Despite these benefits, sequential evaporation has inherent limitations. The interdiffusion of AgI and BiI_3_ layers during post‐deposition annealing must be carefully optimized to ensure complete reaction and phase formation. If the annealing temperature is too low, the conversion may remain incomplete, leading to poor crystallinity or residual precursor phases. Conversely, excessive annealing temperatures can induce thermal decomposition of the halide compounds. Additionally, as the film thickness increases, achieving uniform interdiffusion throughout the entire layer becomes increasingly challenging. Inadequate mixing across the interface may result in inhomogeneous phase formation, elevated defect densities, or residual compositional gradients.

### 4.4 Single‐Source Thermal Ablation

Single‐source thermal ablation (SSTA) is an emerging vacuum‐based deposition technique that offers a simplified route for fabricating Ag–Bi–I rudorffite films using a single, pre‐mixed source.^[^ ^38^ ^]^ As illustrated in Figure 3l, the method relies on flash‐heating a solid precursor mixture containing AgI and BiI_3_, which rapidly sublimes and deposits as a thin film.

Recent work by Nasi et al. demonstrated the feasibility of using SSTA to grow Ag–Bi–I absorbers with varying Ag/Bi ratios (0.5 to 2.0), all of which exhibited a dominant rhombohedral Ag_2_BiI_5_‐like phase. However, Bi‐rich precursors (Ag/Bi ≤ 1.0) led to the unintended incorporation of unreacted BiI_3_ as a secondary phase. Figure 3m,n shows platelet‐like BiI_3_ domains on the surface of Ag/Bi = 1.0 films, as confirmed by high‐resolution transmission electron microscopy (HRTEM), which resolved the (003) lattice fringes of the rhombohedral BiI_3_ phase.

These platelets are believed to arise from surface recrystallization during transient heating in vacuum, highlighting the sensitivity of SSTA to both thermal parameters and precursor composition. Their presence is detrimental to photovoltaic performance, likely disrupting charge transport pathways at the absorber–HTL interface. As shown in the *J–V* characteristics (Figure 3o), solar cells fabricated from Bi‐rich films (Ag/Bi = 0.5 or 1.0) exhibited reduced *J*_SC_ and *V*_OC_, whereas Ag‐rich films (Ag/Bi = 2.0) yielded the best performance: *J*_SC_ = 3.1 mA cm^−2^, *V*_OC_ = 0.71 V, and PCE = 1.0%. These findings underscore the importance of suppressing BiI_3_ formation and optimizing the Ag/Bi ratio to achieve phase‐pure Ag_2_BiI_5_ absorbers with competitive device performance via the SSTA method.

In this study, the precursor for SSTA was prepared by drying a solution of AgI and BiI_3_ directly in the deposition boat, rather than using a pre‐synthesized single‐phase powder.^[^ ^34^ ^]^ This approach simplifies processing and enables rapid compositional screening, but may lead to incomplete reaction or the formation of residual secondary phases, such as BiI_3_ platelets observed in Bi‐rich films. Nonetheless, films derived from Ag‐rich precursors yielded solar cells with efficiencies ≈1%, comparable to those achieved via dual‐source evaporation, despite the absence of interface engineering or device optimization. These results suggest that SSTA offers a practical and scalable route for fabricating Rudorffite absorbers, provided that precursor composition is carefully controlled.

Overall, vacuum‐based deposition techniques enable the formation of clean interfaces, precise thickness control, and excellent compatibility with large‐area thin‐film processing—features that are particularly advantageous for producing uniform, well‐controlled, and semi‐transparent absorbers. Compared with solution processing, vapor‐phase routes offer superior scalability, although their crystallinity and carrier mobility can be somewhat lower than those of optimized solution‐grown films. Continued optimization of growth dynamics is expected to further enhance performance and demonstrate the practical potential of vapor‐phase methods for high‐quality Rudorffite thin films.

## 5 Rudorffite‐Based IPVs

### 5.1 Indoor Suitability of Ag–Bi–I Rudorffites

Building on the processing and material insights discussed above, the potential of Ag–Bi–I rudorffites for indoor photovoltaics has attracted increasing attention in recent years. This is largely due to their wide bandgap and low‐toxicity composition, which makes them highly suitable for IPV applications. Indoor lighting conditions differ significantly from outdoor sunlight: the irradiance is much lower—typically 100–1000 lx in office or home environments—corresponding to only 0.02–0.35 mW cm^−2^ of usable optical power, compared to 100 mW cm^−2^ under standard 1 sun conditions. Moreover, the spectral distribution of indoor light is shifted, as modern light sources such as white LEDs and fluorescent lamps emit primarily in the visible range (400–700 nm) with minimal infrared content. In this context, wide‐bandgap semiconductors (1.8–2.0 eV) are advantageous, as their absorption characteristics align well with the indoor spectrum. Rudorffite‐type Ag–Bi–I compounds, which exhibit strong visible‐light absorption and appropriate band gaps, are thus considered promising candidates for IPVs. Reflecting this potential, recent studies have increasingly focused on evaluating these materials under indoor illumination.

### 5.2 Record Indoor Performance with Planar Architecture

**Figure 4:**
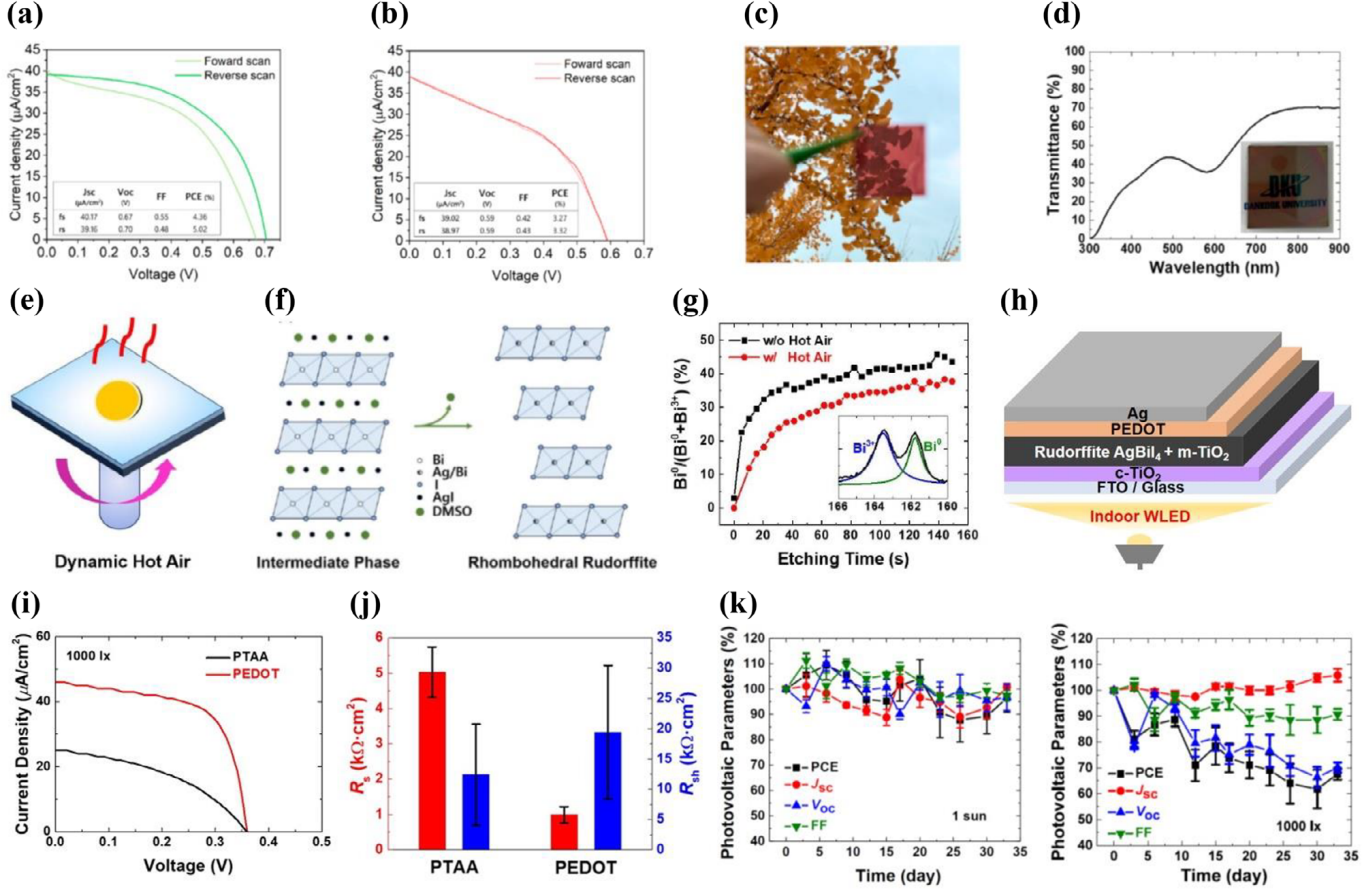
Rudorffite‐Based IPVs. a–c) *J–V* curves of Ag_2_BiI_5_‐based devices under 1000 lx LED illumination, fabricated using a precursor composition of 2AgI:1.5BiI_3_ with concentrations of a) 0.6 m and b) 0.3 m; c) photograph of the semi‐transparent Ag_2_BiI_5_ film prepared under the 0.3 m condition. a–c) Reproduced with permission.^[^ ^13^ ^]^ copyright 2025, ACS Publications. d) Optical transmittance spectrum and inset photograph of a PEDOT/AgBiI_4_/c‐TiO_2_ thin film on ITO‐coated glass. Reproduced with permission.^[^ ^37^ ^]^ Copyright 2024, ACS Publications. e–g) e) Schematic of the hot‐air processing; f) crystallographic transformation from the BiI_3_–DMSO–AgI intermediate to the rhombohedral AgBiI_4_ phase upon DMSO release; g) integrated intensity ratios of Bi⁰/(Bi⁰ + Bi^3^⁺) with and without hot‐air processing at various etching times. Bi 4f_5_⁄_2_ states were deconvoluted using two Lorentzian functions (inset). e–g) Reproduced with permission.^[^ ^39^ ^]^ Copyright 2025, ACS Publications. h–k) h) Device architecture; i) *J–V* curves of AgBiI_4_‐based devices with different HTMs under 1000 lx; j) *R*_s_ and *R*_sh_ estimated from the slope at the *J–V* curve intercepts; k) stability of unencapsulated devices stored in ambient air (15–27 °C, 23–37% RH), measured under 1 sun and 1000 lx LED illumination. h–k) Reproduced with permission.^[^ ^40^ ^]^ Copyright 2024, ACS Publications.

A notable milestone is the report of a 5% PCE under 1000 lx LED illumination for an Ag_2_BiI_5_‐based device (**Figure** **4**a).^[^ ^13^ ^]^ This performance was achieved through precise control of precursor composition and film thickness, enabling enhanced light harvesting under low‐light conditions. In contrast, the same device exhibited a significantly lower PCE (≈1%) under 1‐sun illumination, primarily due to spectral mismatch and increased bimolecular recombination at higher carrier densities. The demonstration of 5% indoor PCE marks a significant advance in validating the potential of Pb‐free rudorffite absorbers for IPV applications, as indoor spectra more closely align with their absorption range and thermalization losses are minimized.

In addition to composition and thickness, device architecture plays a critical role in determining indoor photovoltaic performance. In the aforementioned study, planar Ag_2_BiI_5_ devices achieved PCEs of up to 5% at 1000 lx, clearly outperforming earlier mesoporous counterparts that lacked comparable improvements in film quality. This result indicates that as material crystallinity and morphology are optimized, the reliance on mesoporous scaffolds may become less necessary—particularly under low‐light conditions. While mesoporous structures can facilitate charge extraction by increasing the interfacial surface area, they also introduce additional interfaces that may serve as recombination sites. Under reduced irradiance, such interfacial recombination becomes more pronounced and can offset the benefits of enhanced carrier extraction. As such, planar architectures, when combined with high‐quality film processing, may provide a more effective pathway toward high‐efficiency rudorffite‐based IPVs.

### 5.3 Semi‐Transparent Films for IPV Integration

Even semi‐transparent rudorffite films, which allow partial transmission of incident light, have demonstrated meaningful performance under indoor illumination. For instance, a solution‐processed Ag_2_BiI_5_ device with an absorber thickness below 100 nm achieved a PCE of 3.3% at 1000 lx (Figure 4b,c).^[^ ^13^ ^]^ Additionally, a semi‐transparent AgBiI_4_ film, fabricated via sequential evaporation of AgI and BiI_3_ with a thickness of 65 nm, was incorporated into a photovoltaic device that exhibited a PCE of 2.1% under 1000 lx LED illumination (Figure 4d).^[^ ^37^ ^]^ In the context of IPVs, the development of efficient semi‐transparent devices is particularly attractive, as they can be integrated into windows or other transparent surfaces to harvest energy without significantly compromising visible light transmittance.

### 5.4 Hot‐Air Crystallization for *V*_OC_ and Bi^0^ Suppression

Ag–Bi–I compounds have wide band gaps that, in principle, can support high *V*_OC_—an advantage for indoor photovoltaics where maintaining adequate voltage under low‐light conditions is critical. Under such illumination, however, trap‐assisted recombination and leakage currents become more pronounced, making defect suppression essential.

Hot‐air treatment during film formation addresses these issues by modulating crystallization and suppressing defect formation.^[^ ^39^ ^]^ Notably, it reduces the presence of metallic Bi⁰, which acts as both a recombination center and a leakage pathway. As shown in Figure 4e–g, hot‐air‐assisted processing facilitates the controlled conversion of BiI_3_–DMSO–AgI adducts into the rhombohedral AgBiI_4_ phase by promoting uniform solvent evaporation and gradual adduct decomposition. This stabilizes Bi^3^⁺ and suppresses Bi⁰ formation. XPS confirms a significant reduction of Bi⁰ across the film depth. Since Bi⁰ introduces conductive paths and trap states, its suppression lowers leakage current and recombination losses. Overall, hot‐air‐assisted crystallization provides a scalable and effective route to enhance *V*_OC_ and structural quality in Pb‐free rudorffite IPVs.

### 5.5 Interface Engineering through HTM Selection

The choice of charge transport layers plays a pivotal role in the performance of rudorffite devices. As shown in Figure 4h, most adopt a conventional *n–i–p* architecture, consisting of a TiO_2_ electron transport material beneath the AgBiI_4_ absorber and a hole transport material (HTM) on top. Doped organic HTMs—such as poly[bis(4‐phenyl)(2,4,6‐trimethylphenyl)amine] (PTAA)—are known to cause interfacial degradation, as additives like Li‐TFSI or tBP can chemically interact with Ag–Bi–I films through diffusion or reaction. To avoid this, additive‐free HTMs such as undoped PTAA have been used, albeit with limited conductivity.

A more recent strategy introduced water‐free PEDOT as an alternative HTM.^[^ ^40^ ^]^ Unlike doped counterparts, this formulation avoids chemical damage during deposition while offering higher conductivity and improved interfacial morphology. As shown in Figure 4i, PEDOT‐based devices exhibited significantly higher current densities under 1000 lx illumination than PTAA‐based controls. Furthermore, Figure 4j shows that PEDOT reduced series resistance (*R*_s_) and increased shunt resistance (*R*_sh_), thereby enhancing charge extraction and suppressing recombination. These results underscore the importance of HTM selection in interface engineering and highlight its role in maximizing the efficiency of Pb‐free rudorffite IPVs.

### 5.6 Air Stability Assessed under Indoor Illumination

Device stability in ambient air is a critical factor for realizing practical IPV applications. Several studies have reported that Rudorffite‐based devices exhibit remarkable air stability even without encapsulation. **Table** **1** summarizes representative Ag–Bi–I rudorffite absorbers and their indoor photovoltaic performance metrics, including stability data obtained from unencapsulated samples stored under dark ambient conditions. Notably, the degree of stability strongly depends on the illumination intensity during testing. For instance, unencapsulated AgBiI_4_ devices fabricated via hot‐air‐assisted processing retained ≈89% of their initial PCE after 35 days under ambient conditions when measured at 1000 lx.^[^ ^39^ ^]^ In contrast, the same devices exhibited less degradation—retaining ≈94% of their initial PCE—when tested under 1 sun illumination. Similarly, PEDOT‐based AgBiI_4_ devices showed excellent air stability under 1 sun, maintaining nearly unchanged efficiency for one month, whereas under 1000 lx illumination, a ≈33% reduction in PCE was observed over the same period (Figure 4k).^[^ ^40^ ^]^

**Table 1:** Summary of reported Ag–Bi–I rudorffite absorbers for IPV applications. All PCE and *𝑉*_OC_ values correspond to champion devices. “Ambient Stability” denotes the retained efficiency ratio of unencapsulated samples stored under dark air conditions.

Composition	Structure	Bandgap [eV]	Fabrication Route	Illumination Condition	*V*_OC_ [V]	PCE [%]	Ambient Stability	Reference
AgBi_2_I_5_	Rhombo hedral	1.89	Spin Coating	1000 lx LED	0.70	5.02	‐	[13]
AgBiI_4_	Rhombo hedral	1.94	Spin Coating	1000 lx LED (6500 K)	0.36	3.16	67.7% after 33 Days	[40]
AgBiI_4_	Rhombo hedral	1.97	Hot‐Air‐Assisted Spin Coating	1000 lx LED (6500 K)	0.35	3.32	88.9% after 35 Days	[39]
AgBiI_4_	Rhombo hedral	1.89	Vacuum Evaporation	1000 lx LED (6500 K)	0.35	2.12	48.4% after 34 Days	[37]

These findings underscore the importance of conducting stability assessments under indoor‐relevant illumination, as results obtained under 1 sun cannot be directly extrapolated to low‐light environments. Although rudorffite devices already demonstrate promising intrinsic stability, encapsulation would further enhance their durability. Nonetheless, understanding and mitigating the accelerated degradation observed under low‐light conditions will be critical to ensuring the long‐term reliability of IPV devices based on these absorbers.

Recent developments in rudorffite IPVs indicate that Ag–Bi–I‐based materials are steadily advancing toward practical indoor applications, driven by ongoing improvements in fabrication methods and device design. For instance, devices achieving ≈5% efficiency under 1000 lx LED illumination can deliver ≈16 µW cm^−2^ of electrical power—sufficient to trickle‐charge small batteries or directly power ultra‐low‐energy electronics such as Bluetooth Low Energy (BLE) beacons, sensors, and digital displays. The wide bandgap of Ag–Bi–I absorbers aligns well with the visible‐rich indoor spectrum, while their Pb‐free composition ensures environmentally safe operation. Continued progress in material quality, interface engineering, and compositional tuning will further enhance their efficiency and durability, paving the way for self‐sustaining indoor energy‐harvesting applications.

## 6 Conclusion and Future Perspectives

Rudorffite silver–bismuth iodides represent a new class of eco‐friendly, wide‐bandgap absorbers with clear potential for indoor photovoltaics. Devices have achieved 5% PCE under 1000 lx LED illumination, exhibited *V*_OC_ near 0.8 V under 1 sun, and maintained stability for several weeks without encapsulation. This rapid improvement reflects advances in controlling crystal growth, suppressing metallic Bi⁰ formation, optimizing device architectures, and tailoring interfaces—particularly through the engineering of HTMs—for low‐light operation.

Despite this progress, key challenges remain:

1. Intrinsic limitations such as strong carrier localization and deep defect states reduce carrier lifetimes and limit photovoltaic performance.
2. Interface losses arising from suboptimal conductivity, charge extraction, or energy‐level alignment in transport layers continue to affect overall device output characteristics.
3. Scalability challenges in both solution and vapor processing must be resolved to ensure uniform, large‐area fabrication.
4. Long‐term durability under realistic indoor operation requires a deeper understanding of low‐light degradation pathways and the implementation of effective encapsulation strategies.

Future research should move beyond empirical optimization toward a mechanistic understanding of the structure–property relationships governing Ag–Bi–I rudorffites. In particular, theoretical modeling and defect‐state analyses, supported by first‐principles and polaronic calculations, could provide microscopic insights into the origins of non‐radiative losses. Complementary experimental studies, including temperature‐dependent and time‐resolved spectroscopies, could help to correlate theoretical predictions with carrier localization and recombination dynamics. On the materials side, compositional and strain engineering may help suppress carrier self‐trapping and promote electronic delocalization, while interface and band‐alignment engineering can further enhance charge extraction and long‐term stability.

In a broader materials context, within the family of lead‐free halide absorbers, Rudorffite Ag–Bi–I compounds achieve up to ≈5% indoor PCE and exhibit intrinsic thermal and ambient stability, with photodegradation primarily driven by synergistic light–air effects under high‐energy illumination. Under low‐intensity, visible‐rich indoor conditions, they remain structurally robust. In contrast, Cs_2_AgBi_2_I_9_ and CsMAFA–Sb:Bi have recently attained higher efficiencies (7.6% and 10.1%, respectively) but rely on more complex compositions, which could limit their compatibility with vapor‐based processing.^[^ ^41^, ^42^ ^]^ Considering both intrinsic material stability and manufacturability, rudorffite absorbers are well positioned to become competitive candidates for scalable, vapor‐deposited lead‐free indoor photovoltaics; continued improvements in efficiency will further strengthen their competitiveness.

Finally, integrating these advances into device design strategies and scalable fabrication schemes will be crucial for translating laboratory‐scale performance into practical, high‐efficiency indoor photovoltaic modules. Semi‐transparent and flexible form factors could further expand application opportunities. Achieving PCEs above 10% is a realistic near‐term goal, building on lessons from perovskite photovoltaics but without the environmental burden of Pb. With continued interdisciplinary effort, rudorffite‐based IPVs could evolve from laboratory prototypes to robust, scalable, and sustainable power sources for the next generation of connected devices.

## Conflict of Interest

The authors declare no conflict of interest.
